# Evaluation of root canal morphology of maxillary molars using cone beam computed tomography

**DOI:** 10.12669/pjms.312.6753

**Published:** 2015

**Authors:** Mothanna Alrahabi, Muhammad Sohail Zafar

**Affiliations:** 1 Mothanna Alrahabi, Department of Restorative Dentistry, College of Dentistry, Taibah University, Al Madinah Al Munawwarah, Saudi Arabia; 2 Muhammad Sohail Zafar, Department of Restorative Dentistry, College of Dentistry, Taibah University, Al Madinah Al Munawwarah, Saudi Arabia

**Keywords:** 1^st^ Molar, Endodontics, Vertucci’s classification

## Abstract

**Objectives:**

The success of endodontic treatment is based on cleaning and shaping of the root canals. The root canals have complex morphology and wide individual variations. The objective of this study was to analyze root canals morphology and existence of extra canals in maxillary molars in Saudi subpopulation.

**Methods:**

Freshly extracted maxillary first molars (n=100) were included in this study. All teeth were examined for morphology of roots, root canals and apical foramen by Cone Beam Computed Tomography (CBCT). The root canals configuration was classified using Vertucci’s classification.

**Results:**

The majority of maxillary first molars (94%) were having three distinctly separated roots and 6% had four roots. Palatal and distobuccal roots were observed to contain one root canal (100%) and Vertucci’s type I configuration. The mesiobuccal root had one (29.4%; type I) or two canals (70.6%; type II, III or IV).

**Conclusions:**

The occurrence of second canal in the mesiobuccal root of upper first molar is very much likely (>70%). The mesiobuccal roots are more likely to have Vertucci’s type I or II configuration (>76%). The palatal and distobuccal roots always have a Vertucci’s type I canal configuration.

## INTRODUCTION

The success of endodontic treatment is keenly correlated to the exploration of the entire root canal system, thorough cleansing (mechanical as well as chemical) followed by obturation of prepared root canal system using inert filling materials and a sealant.^1^ In order to achieve this goal, it is crucial to detect each and every canal inside the roots. Any existing root canals that remain undetected by the operator during the entire course of endodontic treatment are a major threat to the failure of treatment.^2^ Hence the detailed knowledge of root canals morphology is essential. Considering the vast individual, genetic and ethnic variations, the clinicians must look for extra canals.

The details of human tooth morphology were published by GV Black in 1902.^3^ Each human tooth has its unique roots anatomy and has been studied in details.^1^ For example, mesiobuccal root of maxillary 1^st^ molar has been reported to have two distinct canals since 1925.^4^ The first molar is the earliest permanent tooth to appear in the oral cavity exposing it for decay and in need of endodontic treatment. A number of studies have been published^5^-^7^ regarding maxillary first molar root canals morphology using various ethnic groups, methods, and approaches. Frequently utilized methods to study the root canal morphology are using staining solutions,^1^ radiographic techniques^8^,^9^ and more recently introduced cone beam computed tomography.^10^ CBCT is a technique that uses a specific beam to produce three dimensional images to reveal anatomic details precisely.^11^ The key advantages of using CBCT are that it is non-invasive and permits 3-D reconstruction of the root canals.^12^ Root morphology and properties of human tooth tissues differ among various ethnic populations.^13^ There is little scientific literature available for root morphology of various teeth in Saudi populations. The aim of the current research was to describe the variations in the root canal anatomy of maxillary first molar in residents of Al-Madinah Al-Munuawarrah, Saudi Arabia using cone beam computed tomography (CBCT).

## METHODS

Freshly extracted maxillary permanent first molars (from residents of Madinah Munawarah, Saudi Arabia; age; 20-60 years) were included in this study (n=100). The patient’s gender and reason for extraction was not recorded. All teeth were inspected carefully to ensure the presence of intact roots with mature apices. Teeth with incomplete, broken or resorbed roots or obturated canals were excluded from the study. In order to remove any debris or attached tissue remnants, all teeth were cleaned and disinfected using 5% NaOCl solution for a day and washed using vigorous amount of ultrapure water. Teeth were kept in the normal saline medium while waiting for further experimentation. The study design was approved by Taibah University, College of dentistry research ethics committee (TUCD-REC-291014).

CBCT acquisition of specimens was conducted using Kodak 9000c 3-D system. The extraoral imaging machine and 3-DKodak Dental Imaging software (KDIS) version 1.3 were used following manufacturer’s guidelines. The image capturing parameters were set at 80 kV, exposure time of 30 seconds at 5.0 mA current. A 14-bit grey scalevoxel size (76×76×76μm) was used. The whole CBCT imaging protocol was carried by a registered dental radiologist following the ALARP [As Low As Reasonably Practicable] protocol.

The CBCT data was analyzed using an Imaging software (CS 3D; Care stream Health, Inc., 2011) and a 21 inches flat screen computer monitor with a resolution of 1440 × 900 pixels (Samsung Seoul, Korea). The contrast function was regulated and the magnifying device was activated when required. The X and Y cursors were used for horizontal and vertical orientation of CBCT images of teeth. In order to survey the anatomy of the roots and canals of maxillary molars from the axial plane, the Z cursor was moved slowly (1 mm interval) in cervico-apex direction. All of the images were assessed separately by two operators and any disagreement was discussed until a consensus was reached.

**Fig.1:**
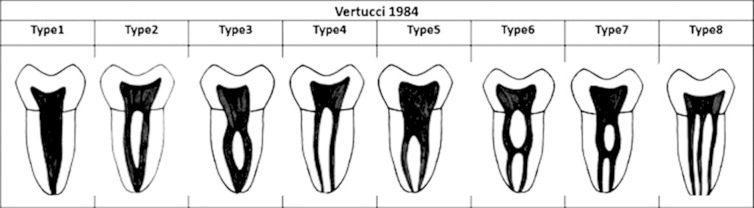
Vertucci’s (1984) criteria for the classification of root canal morphology.

All maxillary molar images were analyzed carefully for roots (number), configuration and number of canals and apical foramina per root. In order to classify the root canal morphology, Vertucci’s^1^ classification (Fig.1) was used as a reference. Data interpretation was performed using SPSS software (version 20) and appropriate statistical tests were performed (p<0.05).

## RESULTS

**Fig.2:**
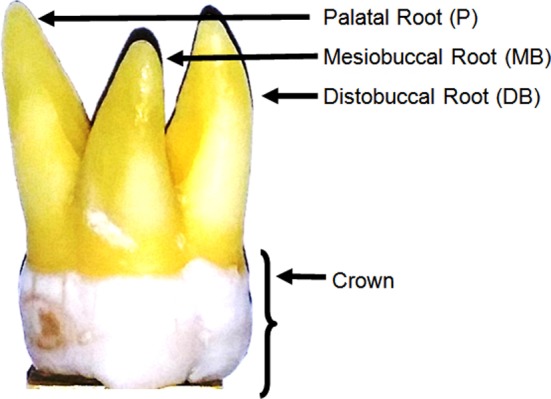
External anatomy of a typical maxillary molar with three diverging roots (mesiobuccal view).

**Fig.3:**
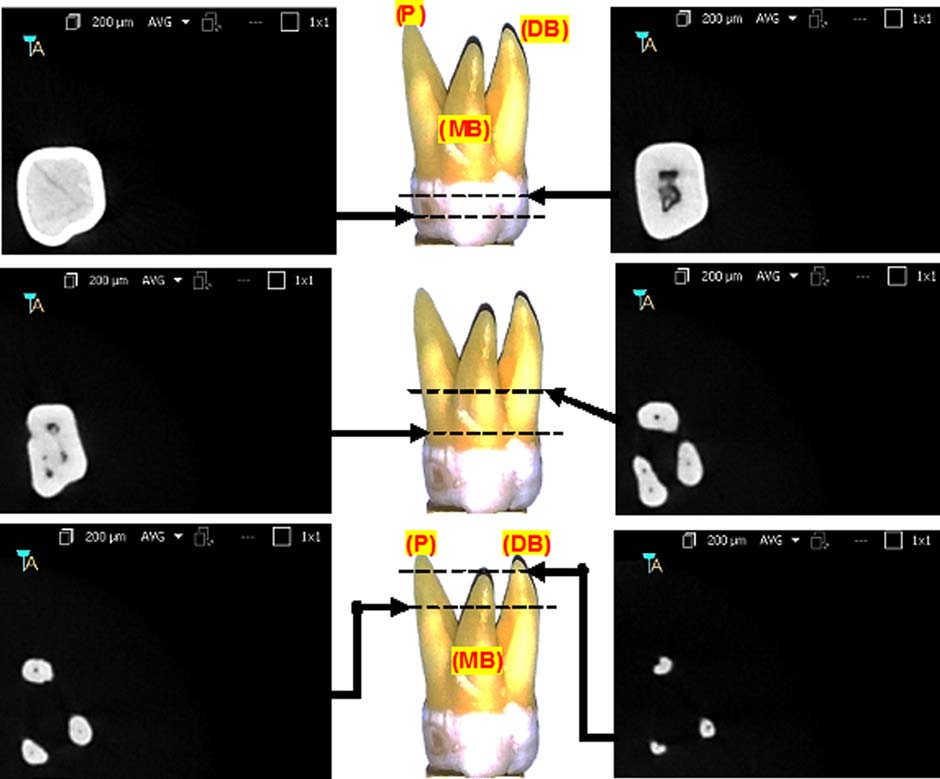
Internal anatomy of a typical maxillary 1^st^ molar (three roots) using CBCT at various anatomical levels.

**Fig.4:**
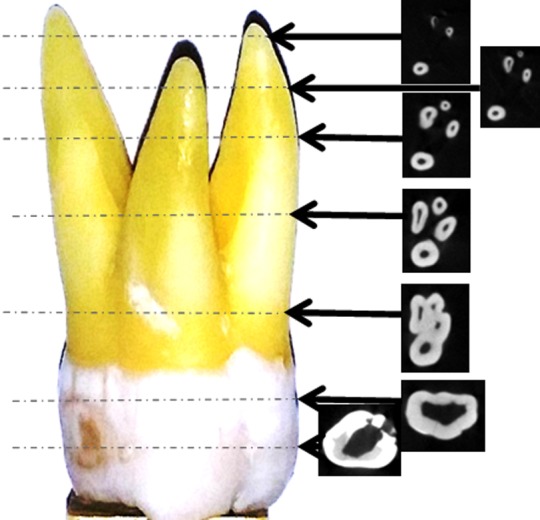
Series of CBCT images of a rare (four rooted) maxillary 1^st^ molar representing the internal anatomy and anatomical variations at various levels.

The physical and radiological examination showed that all teeth included in this study were multi-rooted containing at least three separated roots. The external morphology of typical maxillary first molar roots has been shown in Fig.2. The CBCT images of the same maxillary first molar tooth at various cervico-apical levels have been represented in Fig.3. The majority of teeth were having three distinctly separated roots (94%) followed by four (two palatal) roots (6%). The representative CBCT images of the four rooted maxillary first molar at various cervico-apical levels have been shown in Fig.4.

All roots (palatal and distobuccal) were observed to contain one root canal (100%) except the mesiobuccal root where single root canal was observed in 29.6% of maxillary molars (Table-I). The occurrence of two canals (mesiobuccal and mesiopalatal) in the mesiobuccal root was observed in 70.6% in the selected population.

**Table-I:** Analysis of maxillary first molar root canal morphology.

*Number of roots*				
One	Two	Three	Four
0 %	0%	94%	6%
*Number of canals*				
	One	Two
Palatal root	100%	0 %
Distobuccal root	100%	0%
Mesiobuccal Root	29.4%	70.6%

The classification and canal configurations of the maxillary first molar roots according to Vertucci’s criteria (Fig.1) have been given in Table-II. The root canal configuration for palatal roots and distobuccal roots was very straight forward. Vertucci’s class I configuration was observed in all (100%) of palatal and distobuccal roots (Table-II).

**Table-II:** Classification of root canal morphology in maxillary first molar using Vertucci’s criteria.

TYPE								
	I	II	III	IV	V	VI	VII	VIII
Palatal root	100%	0	0	0	0	0	0	0
Distobuccal root	100%	0	0	0	0	0	0	0
Mesiobuccal root	29.4%	47%	11.8%	11.8%	0	0	0	0

The morphology of mesiobuccal roots remains relatively complex and various types of Vertucci’s root canal configuration types were observed. Vertucci’s type II was found most frequently (47%) followed by type 1 (29.4%) and type III and IV (11.8% each). Other types of Vertucci’s classification (types V, VI, VII and VIII) were not observed at all in any root of maxillary first molars (Table-II).

## DISCUSSION

The significance of knowledge about the anatomic morphology of the maxillary molars and possible variations is vital for successful endodontic therapy and cannot be denied. The current study provides a detailed report on the morphology of root canals of maxillary 1^st^ molars in Saudi Arabian population using CBCT. It is largely recognized that the most frequent pattern of the permanent maxillary 1^st^ molar is comprised of three separated roots.^14^ This study has reported similar results as 94% of cases with three separate roots and only 6% with four separate roots. These finding are in a very close agreement with other studies; Barrett^15^ used the sectioning method to study the anatomy and reported three roots of maxillary first molar in more than 90% teeth. Thomas et al.^16^ examined maxillary molars radiographically using radiopaque gels as canal filler and reported three roots in 94.40% molars. A higher percentage (97.60%) was reported by Al-Shalabi et al.^17^ In the current study, we found 6% of maxillary first molars having four roots. It is obvious that root morphological anomalies are rare however exists in most populations. For example certain studies^18^ have reported four roots (2 palatal), or five roots,^18^ two root canals in a single palatal root^19^ or even three canals.^20^ In addition, incidence of C-shaped canals and root fusion have also been reported.^21^

The main concern for endodontists remains the number of canals inside each root. This study has reported palatal and distobuccal roots contain single canal (100% of cases). The incidence of two palatal canals is very rare.^22^ Cleghorn et al.^5^ has conducted a comprehensive review on the anatomy of the permanent maxillary 1^st^ molar. All inclusive studies in this review analyzed more than 8400 teeth collectively and reported very rare incidence of two palatal roots/canal.^5^ Similar findings have been reported for the distal root, one canal in (100%) of cases in the current study and many other studies^1^,^19^ as well as in Kuwaiti population.^23^ However, a very low incidence (0.1-4%) of two or more canals has been reported.^16^,^17^

The mesiobuccal root is the main focus of morphological studies as the incidence of more than one canal is significantly high and a wide range of variations has been reported.^24^ In our study the mesiobuccal root contained single canal in 29.4% and two canals in 70.6% of cases. The incidence of once canal in mesiobuccal root is comparatively high in Saudi subpopulation. The highest incidence of one canal (75%) has been reported by Woelfel et al.^25^ The reported incidence of two or more canals varies widely; 25%^25^, 55%^1^, 58%^8^, 73.6%^16^ and 78%.^17^ There is not even a single study denying the existence of second canal maxillary first molars.^5^ This is very clear from the above discussion that the internal anatomy of maxillary 1^st^ molar particularly the mesiobuccal root has never been straight forward. There is a high frequency of two or more canals system in the mesiobuccal root and diverges broadly based on factors such as racial ethnicity, population and configuration. The configuration of root canals make it further complicated as six different types of configuration has been classified by Vertucci’s.^1^ In the current study, three configuration types (Vertucci’s type II, III and III) were observed for two canals and only type I was observed for single canal roots.

## CONCLUSIONS

Within the limitations of this study, the following conclusions can be drawn:

1. The maxillary first molar was found a three separated rooted tooth.
2. The incidence of two or four roots or fused roots was very rare.
3. The occurrence of second (mesiopalatal) canal in the maxillary 1^st^ molar mesiobuccal root was very much likely (> 70%).
4. The mesiobuccal roots were more likely to have Vertucci’s type I or II canal configuration.
